# The Effects of Plant-Associated Bacterial Exopolysaccharides on Plant Abiotic Stress Tolerance

**DOI:** 10.3390/metabo11060337

**Published:** 2021-05-24

**Authors:** Rafael J. L. Morcillo, Maximino Manzanera

**Affiliations:** Institute for Water Research, Department of Microbiology, University of Granada, 18003 Granada, Spain; rafaelmorcillo@ugr.es

**Keywords:** exopolysaccharides, abiotic stress, PGPR, salinity, drought, heavy metal, heat stress, cold

## Abstract

Plant growth-promoting rhizobacteria (PGPR) are beneficial soil microorganisms that can stimulate plant growth and increase tolerance to biotic and abiotic stresses. Some PGPR are capable of secreting exopolysaccharides (EPS) to protect themselves and, consequently, their plant hosts against environmental fluctuations and other abiotic stresses such as drought, salinity, or heavy metal pollution. This review focuses on the enhancement of plant abiotic stress tolerance by bacterial EPS. We provide a comprehensive summary of the mechanisms through EPS to alleviate plant abiotic stress tolerance, including salinity, drought, temperature, and heavy metal toxicity. Finally, we discuss how these abiotic stresses may affect bacterial EPS production and its role during plant-microbe interactions.

## 1. Introduction

Crop productivity is influenced by nutrient availability, pathogen diseases, and climatic and agronomic factors, such as radiation, temperature, or water quantity and quality. Global climate change directly or indirectly disturbs these factors, especially those related to abiotic aspects, such as water or temperature, threatening food security [1]. In a world with an increasing population and demand for food, it is imperative to ensure high crop yield to overcome present and near-future demands under the effect of climate change. Plant scientists and agronomists have increased crop production through breeding programs and agronomic practices, including high efficient watering systems, or ambient-controlled green houses, in the past decades. Advances in whole genome sequencing have improved breeding programs and permit to find genome variations in wild crop relatives to obtain and select environmentally adapted and climate-resilient crops [2,3]. However, this strategy is limited to species with a high-quality reference genomic sequence available and populations of wild relatives that grow in diverse environments [4], in addition to the technical difficulties that tend to be labor-intense, highly costly, and poorly welcomed by consumers.

Plant-associated microorganisms are adapted to a wide range of environmental conditions and may represent an alternative source of stress alleviation to increase crop yield and condition [5,6]. Among them, plant growth-promoting rhizobacteria (PGPR) are recognized as beneficial soil microorganisms that can stimulate plant growth or increase tolerance to biotic and abiotic stresses. For instance, some PGPR are able to produce and modulate plant hormones to increase biomass [7,8,9] or trigger plant defense [10], enhance root nutrients uptake [11], or ameliorate the effect of salinity [12,13] or drought [14,15,16] through the production of diverse osmolytes.

Many PGPR are able to secrete extracellular polymeric substances or exopolysaccharides (EPS) that form biofilms and facilitate adhesion to the surfaces of plant roots. EPS are a natural blend of polymers of high molecular weight release by bacteria to their environment as a response to some physiological stresses such as salinity [17], temperature [18], or heavy metal pollution [19] to protect these bacteria against the external environmental variations. Therefore, EPS-producing PGPR may play an important role in alleviating abiotic stresses in plants. During the past decades, many studies have focused on the beneficial effects of PGPR to improve plant growth and mitigate biotic and abiotic stresses. However, the role of bacterial EPS to alleviate abiotic stresses and help plants to adapt to their environment is still under explored. This review attempt to describe and analyze the mechanisms through which EPS enhances plant abiotic stress tolerance. In addition, to provide an overview of PGPR EPS-mediated tolerance to abiotic stress, we consider how external environmental changes influence bacterial EPS composition and structure during plant-bacteria interactions and their effect on stress mitigation.

## 2. Salinity

Salinity is one of the most important threat to agriculture that affects over 10% and 25–30% of the total arable and irrigated land, respectively [20,21]. It does not only influence crop productivity but also soil properties and stability. In the current scenario of global climate change, land affected by salinity is rapidly increasing due to different factors: glaciers and ice sheets melting pushes salty water onshore along coastlines, and heat stress caused by climate change diminish groundwater, accumulating salt concentration in soils [22] and the historical use of chemical fertilizers [23,24]. This phenomenon is especially important in coastlands, where saltwater intrusion into aquifers increases soil salinization. Worldwide, about 600 million people currently dwell in coastal regions will be affected by progressive salinization [25]. Moreover, excessive use of agrochemical fertilizers and pesticides also contributes to increasing salinity in soil, decreasing both soil microbial diversity and the relative abundance of microorganisms [26,27].

In terms of agricultural production, moderate salinity (EC 4–8 dS m^−1^) may cause a 50–80% reduction in crop yield depending on the plant species [28,29], representing a serious threat to food security. For instance, wheat yield decreases up to 10% at a low level of salinity (2.5 dS m^−1^) and 50% at moderate salinity (5.5 dS m^−1^), while a 10% decrease in barley yield occurs at 9.8 dS m^−1^ and 50% at 17.5 dS m^−1^ [28].

Plant roots absorb essential nutrients as soluble salts. However, excessive accumulation of salts compromises ion homeostasis and strongly suppresses plant growth and yield. Under salt stress conditions, photosynthesis is drastically affected, and plant resources are relocated to sustain growth. An increase in salt affects chlorophyll content [30,31] and decreases the photosynthetic electron transport activity [31,32], producing a significant impact on photosynthesis. Salinity hampers the redox chemistry of the primary acceptor quinone, which affects the electron transfer between the manganese complex and plastoquinone molecules [30,33], leading to a reactive oxygen species (ROS) oxidative burst that may cause damage to the photosystem II and hinders photosynthesis [33,34]. In addition, this ROS burst also causes direct injury to plant tissues [34,35]. Moreover, salinity affects photosynthesis by lowering carbon assimilation due to CO_2_ diffusion into the chloroplast is reduced as a consequence of the stomata closure produced to maintain the water turgor pressure in the leaf [36]. This causes inhibition of leaf expansion and, therefore, retardation in plant growth. In addition, the gradual accumulation of salts in plant parts damages cell membrane integrity and lowers membrane stability [37,38]. Nonetheless, the ROS level in plant cells is strongly associated with cell membrane integrity, which produces nutrient imbalance and/or ion toxicity [38]. Moreover, ROS generated due to oxidative stress mediated by salinity (and other stresses) may also produce oxidation of proteins, damage to nucleic acids, enzyme inhibition, activation of programmed cell death and eventually cell death [34,39,40].

A low level of salinity may not impact plant growth but rather flower and fruit production, also leading to a yield loss [29]. Plant responses to salt stress include morphological, physiological, and biochemical changes aimed to exclude or restrict the uptake of toxic ions, maintain osmotic balance, and prevent damage to the photosynthetic process [41,42,43]. Certain strains of PGPR associated with plant roots are able to induce these changes through a variety of mechanisms, including plant hormones production and modulation [44,45,46,47], activation of stress-responsive genes [48,49], osmolytes production and osmotic adjustment [12,13], enzyme antioxidant activation [48], the release of volatile organic compounds [50,51] and bacterial exopolysaccharide production [52,53,54].

In plants, salt tolerance depends on the ability to control ion uptake and homeostasis, discriminating between toxic ions, such as Na^+^ and Cl^−^, and essential elements, as K^+^ and NO^3−^ [55]. Bacterial exopolysaccharides (EPS) have the potential to bind cations including Na^+^ [56] and, therefore, limit their uptake by root plants and maintain K^+^/Na^+^ balance. In this sense, several studies have demonstrated the beneficial effect of EPS to alleviate salt stress by sodium chelation in the soil, which makes Na^+^ inaccessible to plant roots (Table 1). For instance, Kasotia et al. [54] described that EPS produced by *Pseudomonas* sp. AK-1 were able to bind free Na^+^ from the soil, making Na^+^ unavailable to soybean and maintain normal plant growth up to 200 mM NaCl. Similarly, co-inoculation of soybean with *Bradyrhizobium japonicum* and *Bacillus subtilis*, which is well-known for increasing EPS production, alleviated the effects of salinity stress by EPS-mediated sodium uptake restriction; while co-inoculation with *Serratia proteomaculans* was less efficient, indicating the importance of EPS production level and nature [57].

Despite the clear effect of bacterial EPS on Na^+^ chelation, few studies have carried out a deeper characterization of the EPS components involved in Na^+^ binding. According to Nunkaew et al. [62], the capacity of EPS produced by *Rhodopseudomonas palustris* (strains TN114 and PP803) to adsorb Na cations from aqueous solution is attributable to a polysaccharide (≈18 kDa) mainly composed of galacturonic acid. However, other strains of the genera unable to produce EPS are affected by high salinity [68,69], highlighting the importance of EPS in salt tolerance.

On the other hand, NaCl also affects EPS and other bacterial metabolites production. An increase in Na^+^ generally results in increased bacterial EPS production, which in turn may enhance Na^+^ chelation, and also change EPS composition [59,60,62,63,66,70]. For example, EPS carbohydrate and protein content increase with salinity. Tewari and Arora [62] observed that an increase in NaCl concentration modifies EPS sugar composition, with the production of different types of sugars, such as rhamnose or trehalose, whose function seems to be related to enhance the tolerance to salinity and water loss. These sugars function as a carbon reservoir, which protects microorganisms from saline stress and fluctuations in water potential by enhancing water retention and regulating the diffusion of carbon sources in the microbial environment [71]. Moreover, NaCl is able to modify the secretion of other important bacterial metabolites, as indole acetic acid or proline, and negatively affects other PGPR attributes, as phosphate solubilizing ability [17]. Anchoring and adsorption of bacteria by plant roots are also impaired by an excessively high concentration of NaCl due to changes in the ionic strength of the surrounding environment of the bacteria that may affect the periplasmic proteins and monosaccharide composition of bacterial EPS [72]. Conversely, several reports have suggested a greater release of carbonaceous materials from roots exposed to salinity [58,73], which can contribute to attract beneficial bacteria and modify the metabolism of the microbiota already present in the rhizosphere.

Restriction in sodium uptake by the roots is not always attributable to the binding of Na^+^ by EPS. Ashraf et al. [52] proposed that amelioration of salt stress on wheat plants by PGPR-producing EPS was probably caused by a reduced passive flow of Na^+^ into the stele due to the higher proportion of the root zones covered with soil sheaths in PGPR-inoculated plants. In this direction, several studies have pointed out that the increased soil adhesion to roots resulting in a higher mass of rhizosphere soil is attributable to bacterial EPS [74,75,76], which has been associated with the content of water-insoluble saccharides in the rhizospheric soil and salinity tolerance [52,53]. Soil aggregation and its structural stability are reduced with the presence of NaCl [77]. However, it has been reported that soil aggregation increases in soils inoculated with PGPR around the roots under high salt concentrations. Biofilm formation and the binding properties of bacterial EPS help the soil particles to stick together and with roots, favoring plant growth under salt stress [60,61,62], although the sticky nature of EPS depends on its composition, special of the type and concentration of sugars, proteins and lipids composition [78,79].

In addition, increasing the EPS production under salt stress leads to biofilm formation [59,65,66], which in turn protects bacteria and their associated plant host against salt stress by retaining a water layer around the cells and improving cell adhesion [80,81]. Nevertheless, NaCl may not affect EPS production and biofilm formation at the same level of salinity. For example, maximum biofilm formation of the bacterial strains *Halomonas meridiana* PAa6, *Kushneria indalinina* HT2, and *Halomonas aquamarina* ST2 is established at 1 M salt concentration, while maximal EPS production occurs below 1 M NaCl [61], indicating that the abundance of EPS is not an indication of biofilm formation. However, its production may influence the biofilm architecture to a variable extent [81] and, therefore, the protector role of biofilms in plants subjected to saline stress.

In agriculture, salts are often added by fertilizers, which excess frequently have a tremendous impact on soil salinization. Among the mineral fertilizers, KCl has the highest impact on soil salinity because potassium is efficiently uptake by plants as a macronutrient, while chloride, which is a micro-nutrient, stays in the soil [23,82]. Therefore, it is plausible to find a high concentration of Cl^−^ in soil not related to NaCl. Similar to Na^+^, high levels of chloride ion may have a negative impact on plant growth and yield. For instance, Cl^−^ disturbs photosynthesis by reducing the efficiency of photosystem II and interacts with the uptake of other anions indispensable for plant growth, such as NO^3-^ and PO_4_^2−^ [83]. It has been shown that plant inoculation with different PGPR strains reduces Cl^-^ uptake and increases plant growth [84,85,86]. Similarly, Abd El-Ghany and Attia [85] reported a decrease in Cl^−^ ion in faba beans inoculated with an EPS-producing *Azotobacter chroococcum* strain in combination with melatonin. However, most of the studied bacterial EPS are negatively charged, due to the dominance of carboxyl and hydroxyl functional groups [87,88,89,90,91], indicating that the decrease in chloride anion concentration in PGPR-inoculated plants might not be related to the adsorption capacity of Cl^−^ by EPS.

Besides Na^+^, EPS generated by rhizobacteria is able to immobilize other cations such as Ca^2+^, Mg^2+,^ and K^+^ ions from salts [52,56], and producing different impacts on plant growth. For instance, Ca^2+^ and K^+^ retention by EPS might reduce the internal level of these cations in plants. However, it is rare to observe a negative effect on plant growth and yield caused by calcium or potassium deficiency mediated by EPS-producer rhizobacteria under non-saline conditions due to the low level of these cations necessary for normal plant growth. In contrast, in saline environments, the competitive effect of Na^+^ may cause Ca^2+^ deficiency in plants [92]. This phenomenon could be enhanced by the retention effect of Na^+^ produced by certain EPS-producer rhizobacteria strains, affecting the Ca^2+^ level and Ca^2+^/Na^+^ ratio in roots. Nonetheless, this alteration seems to be not sufficient to cause Ca^2+^ deficiency in the plant tissues or to affect yields [52].

In conclusion, bacterial exopolysaccharides have a high potential application to ameliorate salt stress in plants due to their capacity to chelate free Na^+^ from the soil, making Na^+^ unavailable to plant crops, enhancing soil aggregation and stability, and supporting biofilm formation that retains water layer around roots and improves cell adhesion. In general terms, salt-tolerant rhizobacteria might maintain normal plant growth up to a concentration of 150–200 mM NaCl, depending on the plant species and the bacterial strain. For instance, *Pseudomonas sp.* AK-1 and *B. amyloliquefaciens* HM6 are able to hold soybean and barley normal growth up to 200 mM NaCl, respectively [54,65], while *B. amyloliquefaciens* SRQ9 does not maintain normal maize growth at 100 mM NaCl [64]. Despite the well-known effect of certain PGPR to alleviate salt stress, little is still known about the physical and chemical properties of EPS that help plants to cope with salt stress, especially in EPS change composition under different levels of salinity.

## 3. Drought

Water deficit caused by climate change represents a significant agricultural threat that limits crop growth and productivity and, therefore, food security. According to the Food and Agriculture Organization of the United Nations (FAO), between 2005 and 2015, eighty-three percent of all drought-caused economic losses will be absorbed by agriculture [93]. Lesk et al. [94] estimated that droughts and extreme heat significantly reduced global cereal production by 9–10% in the past decades, being this phenomenon associated with a reduction in both harvested area and yields. Particularly, the harvested area dropped more than 5% during droughts events produced during the past decades [94], pointing out the constant loss of agricultural soil that is currently occurring. Similar to salt stress, drought does not only influence the growth and productivity of crops but also soil properties and stability. Drought stress changes physico-chemical and biological properties of the rhizosphere that affect soil microbial activity and crop yield [95]. Therefore, maintenance of soil structure is an important feature of sustainable agriculture because it impacts a range of processes influencing crop growth and productivity.

To control water loss and similarly to salt, one of the first responses of plants subjected to water deficit is a stomatal closure, which leads to a disruption in gas exchange and CO_2_ assimilation and, therefore, affects photosynthesis activity [96,97,98]. In addition, drought stress reduces chlorophyll content as a result of pigment photo-oxidation induced by an oxidative burst produced by overproduction in ROS [99], which also affects protein and lipid peroxidation, hampering membrane integrity and stability [39,100]. ROS burst induced by water deficit is produced as a result of the stomatal closure, which increases the incident radiation with respect to the available intracellular CO_2_, disrupting the rate of electron production [101]. Hence, drought-induced stomata close progressively affects photosynthesis, gas exchange, and water use, leading to an inhibition of leaf expansion and, therefore, plant growth and yield.

Several mechanisms have been proposed for PGPR-mediated drought stress tolerance in plants, which include plant hormonal regulation [102,103,104], root morphology modification [105,106], ACC (1-aminocyclopropane-1-carboxylate) deaminase activity [107,108], accumulation of osmolytes and antioxidants [14], volatile organic compounds [50] and EPS production [67,109,110]. For instance, indolacetic acid (IAA) produced by PGPR increases root growth and formation of lateral roots, helping plants to enhance water and nutrient uptake and alleviating drought stress [49,106,111,112]. Similarly, some PGPR strains are able to generate other hormones, such as gibberellin, cytokinin, and abscisic acid, of which production has been related to plant drought protection [103,104,113]. Moreover, PGPR may also modulate internal levels of plant hormones, improving plant tolerance to drought stress [15,102,114,115]. It is particularly interesting the role of bacteria ACC deaminase to reduce the negative effect of ethylene during drought stress in plants [116]. During water deficit, plants synthesize ethylene to regulate plant homeostasis resulting in reduced root and shoot growth [117]. The action of bacterial AAC deaminase degrades plant ethylene reducing the deleterious effect of this hormone, ameliorating plant stress, and promoting plant growth [116].

In addition to hormone production and modulation, PGPR increase antioxidant and osmolytes accumulation in plants during water stress, such as proline, sugars, polyamines, betaines, quaternary ammonium compounds, polyhydric alcohols, and other amino acids [15,49,67,118,119]. These osmolytes counterbalance the osmotic pressure altered by drought stress, protecting and rescue plants from the stress caused by oxidative damage and, therefore, maintaining plant growth. Moreover, inoculation with some PGPR strains modulates the activity of plant antioxidant enzymes such as catalase, ascorbate peroxidase, glutathione peroxidase under drought stress [14,120,121,122]. This modulation reflects the beneficial effect of PGPR application in enhancing drought tolerance of plants by altering the antioxidants activity under water deficit conditions.

Despite the morphological and physiological advances in PGPR-mediated protection to plant drought stress, little is still known about how EPS-producing PGPR mediate physiochemical and hydrological changes in the rhizospheric soil that may impact plant drought stress tolerance. Bacterial EPS and capsular polysaccharides maintain a hydrated microenvironment around the bacteria, reducing water loss, which promotes bacterial survival under drought stress [123,124]. EPS production and composition change with the increase in stress level [125]. For instance, under extreme conditions, such as desiccation, *Azospirillum brasilense* Sp245 produces high molecular weight carbohydrate complexes (lipopolysaccharide-protein and polysaccharide-lipid complexes) that might be responsible for bacterial protection [126]. Encompassing itself with a layer of extracellular polysaccharides containing high water content, the bacteria guarantees a better resistance against desiccation [125] and increases water availability in the rhizosphere. This enhances plant resistance to drought by increasing the time available for plants to make metabolic adjustments to cope with the stress.

EPS and capsular polysaccharides represent the major components of biofilm and participate in cell-cell aggregation that is crucial for bacteria anchoring and adhesion to plant roots [127,128]. Therefore, the absence of EPS affects biofilm formation and root colonization, which may impact drought resistance in plants. To determine the importance of EPS in root colonization to alleviate drought stress in plants, Lu et al. [110] constructed an exopolysaccharide-deficient mutant of *Bacillus amyloliquefaciens* FZB42 lacking the *epsC* gene, a key factor responsible for the production of EPS and biofilm formation. *Arabidopsis thaliana* inoculated with the *epsC* mutant showed a decreased capacity for inducing drought tolerance in plants due to its minor capability to support the formation of biofilm and further colonization of *A. thaliana* roots [110]. Interestingly, colonization of EPS-producing *Pantoea aglomerans* NAS206 appears to increase at the rhizoplane and in root-adhering soil (RAS) but not in bulk soil and under relatively dry conditions in wheat plants [129], indicating the importance of EPS in root colonization and drought stress. The colonization of the wheat rhizosphere by *P. aglomerans* NAS206 was associated with a significant augment of soil aggregation, which maximum effect was observed at 24% average soil water content (matric potential, −0.20 MPa) [129]. Similarly, inoculation of sunflower seeds with *Rhizobium* sp. YAS34 caused a significant increase in RAS per root dry (RAS/RT) mass and soil macropore volume, which was associated with plant growth promotion under both water stress (matric potential, −0.60 MPa) and normal water supply conditions [109]. RAS forms the immediate environment where plants take up water and nutrients for their growth. Increase RAS/RT and porous soil structure favor water retention around the roots and, in consequence, water and nutrient uptake. Moreover, the anionic nature of the exterior polysaccharide layer can help to capture essential minerals and nutrients [125].

The polysaccharides secreted by EPS-producing bacteria may be adsorbed on soil particles and cement them due to the formation of cation bridges, hydrogen bonding, van der Waals forces, and anion adsorption mechanisms between soil particles [130,131,132]. EPS has a highly cross-linked structure that enables it to shrink and swell yet remain saturated across a wide range of matric potentials [133]. For instance, xhantan and alginate polymers from *Xhantomonas sp* and *Azotobacter vinelandii* improve soil aggregate formation [134]. However, the formation of stable aggregates seems to depend on both the nature and the content of organic matter and the type of soil particles [131,132,135,136], and little is known about the rheological properties and thickening agents responsible for soil aggregates formation. One of the few studies that address this aspect of bacterial EPS suggests that the structure of the thickening agents of EPS produced by *Rhizobium* sp. KYGT207 and *Sinorhizobium sp.* YAS34 are composed of glucose, galactose and mannuronic, and glucose, galactose, and glucuronic acid, respectively [136]. Interestingly, Fersiukh et al. [132] have recently demonstrated that silica particles triggered the PGPR production of EPS containing D-glucuronate, which subsequently increases the water holding capacity and osmotic pressure of bacteria biofilm and root colonization, promoting plant growth in drought-stressed environments.

This bacterial contribution to soil aggregation in the vicinity of root surfaces has important consequences in terms of water and mineral uptake. In the rhizosphere, EPS act as a reservoir and a conductor of water to plant roots when bulk soil water is scarce [137]. For instance, the maximum moisture content of the soil has been recorded for the rhizosphere soil inoculated with different PGPR strains in combination with their respective EPS both under drought and unstressed conditions [138]. In this line, Zheng et al. [139] demonstrated that PGPR’s ability to increase water availability to plants is related to a reduction in hydraulic conductivity and soil water evaporation due to changes in the soil structure and physicochemical properties of water (surface tension and viscosity) mediated by the influence of bacterial EPS. Similarly, Deng et al. [140] demonstrated that EPS from *Sinorhizobium meliloti* limit water evaporation at pore throats reducing water loss in soil, using soil micro-models.

Despite the influence of EPS in soil structure and water holding capacity to alleviate drought stress in plants, little is known about the potential direct impact of EPS composition in plant physiology to improve drought resistance. In this sense, Naseem and Bano [138] suggested that differences in the functional groups of bacterial EPS may trigger different plant antioxidant mechanisms to cope with drought stress. For instance, the activity of antioxidant enzyme superoxide dismutase was higher in maize plants inoculated with *Pseudomonas aeruginosa* Pa2, whereas peroxidase activity was greater in inoculated *Alcaligenes faecalis* Af3 plants under drought stress conditions [138].

In conclusion, bacterial EPS have a great potential to alleviate drought stress in plants due to their capacity to improve soil structure and retain soil water, promote bacterial colonization and biofilm formation, and regulate plant response to water deficit, increasing the time available for plants to make metabolic adjustments to drought stress (Table 2). For instance, *P. putida* GAP−45, as well as certain strains of *Bacillus*, are able to maintain normal plant growth up to a maximum matric potential of −0.73 MPa during a period of 4–6 days [14,66,120]. Nevertheless, investigate the rheological properties and composition of the thickening agents of bacterial EPS responsible for soil aggregates formation and water holding would contribute to a better understanding of the mechanisms involved in the reduction in drought stress in plants and, therefore, to find solutions for drought limitation of crop growth and productivity.

## 4. Heavy Metal

Heavy metal accumulation by anthropogenic activities, such as industrialization or modern agricultural practices, causes a wide range of human health, environment, and agricultural problems [144,145]. Heavy metal content in soil depends on the composition and nature of the bedrock; however, in soils for agricultural use, the concentration of these elements can be increased by the addition of various types of substances that contain them to a greater or lesser extent proportion. Thus, heavy metal could be extractable by plants and constitute a serious problem in crop productivity and quality [146]. Plants have evolved a varied range of physiologic, metabolic, and genetic defense strategies to cope with heavy metal toxicity. These strategies are primarily focused on restricting metal uptake from soil to prevent heavy metal entry into plant roots [147,148]. For instance, low-molecular-weight organic acids from root exudates may act as chelating agents to limit heavy metal uptake by plants [149]. In addition, if heavy metals manage to enter inside plant tissues, detoxification and defense antioxidant mechanism are activated [150]. However, despite these defense mechanisms, most plant species suffer from poor growth rate and productivity in a high concentration of heavy metals. This problem can be alleviated by microbial assistance [151,152].

Bacterial mechanisms resistance to heavy metal includes metal exclusion by a permeable barrier or by active export of metal from the cell; detoxification of heavy metals by chemical modifications; and physical sequestration by metal-binding extracellular polymers as exopolysaccharides (EPS) and liposaccharides [19,88,153]. The structure and composition of EPS promote metal ion sequestration by biosorption due to the interaction between positively charged metal ions and negatively charged EPS. The abundance of different carboxyl and hydroxyl functional groups and non-carbohydrate substituents, such as acetamido, amine, sulfhydryl, and carboxyl groups in proteins, are responsible for the anionic feature and negative charge of bacterial EPS [19,88]. Due to these characteristics and other physiological, rheological, and physiochemical properties, bacterial EPS have been widely studied for heavy metal bio-remediation [88], and EPS-producing bacteria have been suggested as assistant agents of plants for heavy metal phytoremediation [152]. However, despite the well-known role of bacteria in heavy metal remediation, little is known about the specific role of EPS during plant-microbe interactions subjected to heavy metal stress.

Some plant growth-promoting rhizobacteria (PGPR) may alleviate metal phytotoxicity and stimulate plant growth directly through the solubilization of mineral nutrients, production of plant growth-promoting substances, and secretion of specific enzymes [154]. For instance, EPS-producing *Bacillus gibsonii* PM11 and *B. xiamenensis* PM14 enhanced nutrient availability and plant growth of *Linum usitatissimum* by minimizing metal-induced stressed conditions [155]. Similarly, three moderate halophiles, *Halobacillus sp*. ADN1, *Halomonas sp*. MAN5, and *Halobacillus sp*. MAN6 were able to retain indole acetic acid and phosphate solubilization capacity in the presence of salinity and heavy metals such as 1 mM cadmium, 0.7 mM nickel, 0.04 mM mercury, and 0.03 mM silver to enhance the root growth of *Sesuvium portulacastrum* [156]. However, a high concentration of heavy metals may affect bacterial plant growth-promoting (PGP) features. A concentration of 100 µM of Al^3+^, Cd^2+^, Cu^2+^, Fe^3+,^ and Ni^2+^ negatively affected auxin production in *Streptomyces spp.* [157], while an increasing trend of Cr (VI) concentration (from 0 to 250 µg/mL) progressively declined indole acetic acid and ammonia production of *Cellulosimicrobium funkei* AR6 but increased EPS production, indicating the role of EPS as a defense mechanism to alleviate heavy metal stress in the bacteria and its host [158]. In this direction, Mukherjee et al. [159] observed that arsenic (2 mM) increases *Halomonas sp*. Exo1 exopolysaccharides production that in turn sequestered the metal, alleviating heavy metal stress in rice and showing a positive feedback mechanism.

Despite of the potential of bacterial EPS to ameliorate heavy metal stress in plants, few studies have investigated the effect of heavy metal in EPS conformation and composition during plant-microbe interactions. EPS characterization through FT-IR and SEM-EDX analyses of *Halomonas sp*. Exo1 EPS revealed that their structure and composition in the presence of arsenic favor metal ion sequestration by biosorption due to the negative charge matrix of the EPS [159]. On the other hand, Cd^2+^ causes a change in EPS conformation to the more planar configuration, which reduces the volume of liquid in the interglobular space next to the bacterial wall to minimize the impact on bacterial cells [160]. These structural changes may affect plant-microbe interactions since EPS structure, and composition affects biofilm formation and root colonization, which, in turn, may impact the role of EPS-producing bacteria to promote plant growth and alleviate biotic and abiotic stresses. For instance, EPS defective mutants of *Sinorhizobium meliloti* are affected on biofilm formation, which is important for cell-cell interactions and surface attachment, and more sensitive to Hg(II) (20 µM) and As(III) (100 µM), indicating the importance of EPS in biofilm-mediated protection to heavy metal stress in legumes [161].

Other abiotic factors might affect plant heavy metal resistance mediated by bacterial EPS. In this sense, salinity can act as an inducer of EPS production of *Halomonas* sp. Exo1, while the addition of arsenic in a salt-amended media augments EPS production, which, in turn, would enhance metal EPS-mediated sequestration and plant heavy metal resistance [159]. This would be especially important in saline soils that frequently chelate heavy metals from the surrounding environment, increasing their phytotoxicity when heavy metal pollution occurs in this type of soil [156].

The role of bacterial EPS in heavy metal bioremediation has been extensively studied [89]. Moreover, PGPR are used to accelerate heavy metal phytoremediation [151]. However, little is known about the effect of heavy metal in EPS composition and, therefore, in its effect on root colonization by EPS-producing PGPR and their beneficial effect on plant growth and health.

## 5. Temperature

Global climate change impacts current and future mean temperature and increases the risk from extreme weather events, including a period of both extreme heat and frost. Heat and cold shocks are physical stresses that directly influence molecular (DNA and proteins) and supramolecular (membranes, chromosomes) structures, affecting plant growth and productivity [162,163]. One of the major consequences of heat and cold stress is the excess generation of ROS, which leads to oxidative stress [164,165], producing the damage of membranes, pigments, proteins, and nucleic acids and, consequently, impairing plant growth and development [166,167]. As with other types of abiotic stresses, heat and cold stress also alter chlorophyll biosynthesis and photosynthesis since both stresses drastically affect chloroplast metabolism and structure. For instance, heat shock disturbs the structural organization of thylakoids and promotes loss of grana stacking and swelling of grana [168,169], while low temperature induces changes in the organization of the photosynthetic apparatus that lead to a decrease in the number of functional PS II reaction centers, loss of light-harvesting Chl and the formation of a large thylakoid protein complex involved in LHC II, PS II, and PS I [170,171]. Even short episodes of both types of stresses can reduce crop yield considerably [172]. Plant responses to heat and cold comprise changes at molecular, physiological, and cellular levels. Among those responses, plants produce compatible solutes, antioxidants, and osmoprotectants substances to organize and protect proteins and cellular structures and to maintain cell turgor by osmotic adjustment [165,173,174,175]. Moreover, cold and heat stresses may also reduce water absorption by plants due to the decrease in their water potential, which can lead to dehydration [172,176]. These responses might be ameliorated by plant-associated bacteria, including PGPR, which can help plants to increase the time available to adapt their metabolism and become acclimatized to cope with heat and cold stresses.

Several studies have explored the role of PGPR to protect crops against high temperatures. For instance, Ali et al. [177] investigated the effect of plant growth-promoting thermotolerant *Pseudomonas putida* strain AKMP7 on the growth of wheat plants to heat stress. Inoculation with *P. putida* AKMP7 improved the levels of cellular metabolites such as proline and reduced membrane injury and the activity of several antioxidant enzymes such as SOD, APX, and CAT under heat stress [177]. Similarly, inoculation of wheat seedlings with *Bacillus amyloliquefaciens*, *Azospirillum brasilense*, *Bacillus safensis*, or *Pseudomonas brassicacearum* reduced the generation of reactive oxygen species and adjust wheat seedlings metabolism to enhance plant resistance to heat stress (40–45 °C) [178,179,180]. However, despite the potential of PGPR to protect crops against high temperatures, very little information is available concerning the improvement of heat stress tolerance using EPS-producing bacteria and the effect of EPS to alleviate heat shocks.

High-temperature influences EPS production of plant-associated bacteria. According to Mukhtar et al. [181], heat stress enhanced EPS production of *Bacillus cereus* and cleavage of 1-aminocyclopropane-1-carboxylate (ACC) of plant inoculated with this strain to significantly reduce the adverse effects of heat (42 °C) on tomato growth. Moreover, elevated temperatures increased EPS and lipopolysaccharides production in *Rhizobium* sp. heat-resistant mutants [18], while *Shinorizobium meliloti* mutants affected in oligomeric proteases complexes *HsIUV* and *ClpXP1* are affected in adaptation to heat stress (45 °C) and produce less quantity of EPS [182]. In this direction, Nishihata et al. [183] observed that inactivation of *phaR*, a transcription factor that controls poly-3-hydroxybutyrate synthesis in *Bradyrhizobium diazoefficiens*, augments EPS production and improves heat stress tolerance. These results suggest a direct effect of EPS to alleviate heat stress in PGPRs, which may be related to a protecting role of the surrounding matrix produced by EPS around the roots that might act as a dampen agent against heat but also improve water retention [125], alleviating the effects of the heat shock in the plant. This idea is in agreement with the results obtained by Ali et al. [184], who observed that several plant-associated beneficial bacteria were resistant to elevated temperatures due to the production of EPS and accumulation of heat shock proteins that probably form a microsheath around microbial cells and roots to protect them from surrounding harsh conditions.

Low temperatures (from 0 to 15 °C) also increase EPS production of plant-associated bacteria. For instance, psychrotolerant *Pseudomonads* from Northwest Himalayas are able to produce higher amounts of EPS under cold conditions than at ambient temperature [185]. Similarly, *Bacillus* sp. CJCL2 and RJGP41 alleviate cold stress in wheat seedlings by producing a thin biofilm structure that enables bacterial cells to survive and secrete specific metabolites under cold stress to protect them and the host plants under cold stress and facilitate root colonization in these conditions [186]. However, an increase in EPS production under cold stress is not a ubiquitous feature in plant-associated bacteria since *Bacillus velenzensis* F2B42, a non-tolerant cold PGPR strain, produce a better structure of biofilm at ambient temperature (25 °C) than cold-tolerant (4 °C) strains but fails to produce it in cold conditions [186].

Cold stress leads to dehydration and osmotic imbalances in plants, which may alter the Na^+^/K^+^ ratio in roots, affecting numerous metabolic enzymatic activities and nutrient uptake [55,176]. However, bacterial EPS can chelate Na^+^ ions restricting sodium uptake by roots [17,52] and, therefore, protecting plants against cold-mediated dehydration.

Heat- and cold-tolerant plant-associated bacteria are significant for agriculture since, in many regions of the world, crops are subjected to heat or cold events, and these bacterial strains are metabolically adapted to these temperature conditions, being able to promote plant growth and health under such conditions. Special attention has EPS-producing plant-associated bacteria, which are capable to physically protect plants, creating a surrounding matrix around the roots that dampens the effect of temperature changes.

## 6. Conclusions

**Figure 1:**
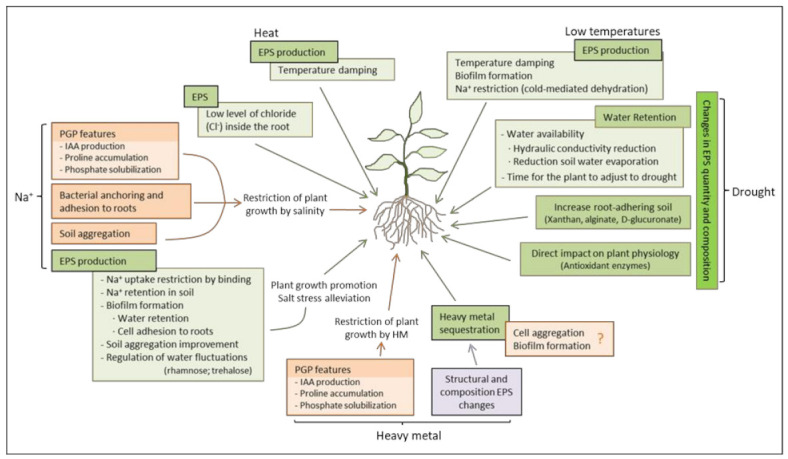
Microbial exopolysaccharides assisted mechanisms to alleviate abiotic stresses in plants. EPS can alleviate sodium-mediated salinity, restricting Na+ uptake by plant roots, improving soil aggregation, regulating water fluctuations, and favoring b.

Bacterial exopolysaccharides (EPS) are a natural blend of polymers of high molecular weight released by bacteria to their environment, which secretion is produced in response to a variety of external stresses, including salinity, drought, heavy metal toxicity, and changes in temperature [125,187]. In addition, EPS are involved in cell-cell aggregation that is crucial for bacteria anchoring and adhesion to plant roots [127,128]. These characteristics make EPS an important factor to exploit in plants’ protection against different types of stresses through bacterial application. In this sense, research on abiotic stress tolerance induced by bacterial EPS in plants has revealed some interesting phenotypes and initial insights into the underlying mechanisms (Figure 1). However, and compared with other well-known characteristics of plant-associated beneficial bacteria, EPS effects on plant abiotic stress amelioration is still underexplored. Especially important is the effect of environmental pressures on EPS composition and structure of plant-associated bacteria and its potential affection on the symbiosis establishment. Therefore, research on EPS should continue to improve the protection of crops from abiotic stresses and to better understand the underlying physiological and molecular mechanisms, which will permit an appropriate application of EPS-producing bacteria to crops to maintain productivity and ensure food security.

## 7. Researched Literature

National Center for Biotechnology Information (NCBI), ISI Web of Knowledge (WoK) from the Institute for Scientific Information (ISI), Scopus, and Google Scholar databases from 1980 to 2020 were searched. Search terms for the general information and the specific abiotic stresses were:

*General information*: ‘exopolysaccharides’, ‘extracellular polysaccharides’, ‘EPS-producing bacteria’, ‘PGPR’, ‘rhizobacteria’, ‘abiotic stress’, ‘biofilm’, and ‘plant stress’.

*Salinity*: ‘salinity’, ‘salt stress’, ‘soil salinity’, ‘hyperosmotic stress’, ‘salinity tolerance’, ‘sodium toxicity’, ‘ion toxicity’, ‘sodium uptake’, ‘chloride toxicity’, ‘salinity by fertilization’, and ‘halophilic bacteria’.

*Drought*: ‘drought’, ‘drought tolerance’, ‘desiccation’, ‘drying stress’, ‘water stress’, ‘osmolytes’, ‘water retention’, and ‘soil aggregation’.

*Heavy metal toxicity*: ‘heavy metal’, ‘heavy metal toxicity’, ‘heavy metal stress’, and ‘remediation of metal contamination’.

*Temperature*: ‘cold stress’, ‘low temperature’, ‘freezing temperature’, ‘heat’, ‘heat stress’, ‘high temperature’, ‘elevated temperature’, and ‘thermoloterance’.

These terms produced a wide range of articles that provide extensive information on stress amelioration in plants by EPS and non-EPS-producing rhizobacteria that were deeply studied and narrowed down by the specific topics to obtain further specific information. Articles were rejected if it was determined from the title and the abstract that the study failed to meet the inclusion criteria. Any ambiguities regarding the application of the selection criteria were resolved through discussions between the researchers involved.

## Figures and Tables

**Table 1:** PGPR’s mechanisms to alleviate salinity stress in plants.

Bacteria	EPS Mechanism	Crop	Reference
*Aeromonas hydrophila/caviae* MAS765 *Bacillus insolitus* MAS17 *Bacillus sp.* MAS617 *Bacillus sp.* MAS620 *Bacillus sp.* MAS820	Restricted Na^+^ uptake by a reduced passive flow of Na+ into the stele due to the higher proportion of root zones covered with soil sheaths in inoculated plants	Wheat	Ashraf et al. [52]
*Bacillus circulans* UBF 26			
*Bacillus polymyxa* UBF 15			
*Bacillus subtilis* *Serratia proteamaculans*	Restricted Na^+^ influx due to free soil Na^+^ binding by EPS	Soybean	Han and Lee [58]
Consortium of Cyanobacteria	Removes Na^+^ from aqueous solution by Na^+^ adsorption	Wheat; Maize; Rice	Arora et al. [59]
*Bacillus licheniformis* SKU3	Restricted Na^+^ uptake in saline and non-saline soil by EPS	Wheat	Upadhyay et al. [17]
*Bacillus pumilus* SKU4			
*Bacillus sp.* SKU5			
*Burkholderia cepacia* SKU6			
*Enterobacter sp.* SKU7			
*Enterobacter sp.* SKU8			
*Microbacterium sp.* SKU9			
*Paenibacillus macerans* SKU10			
*Paenibacillus sp.* SKU11			
*Bacillus coagulans* SKU12			
*Bacillus insolitus* SKU13			
*Oceanobacillus profundus* Pmt2 *Staphylococcus saprophyticus* ST1	Na^+^ chelation. Increased mass of roots adhering soil (RAS) and biofilm stability	*Lens esculenta*	Qurashi and Sabri [60]
*Halomonas variabilis* HT1 *Planococcus rifietoensis* RT4	Enhances soil aggregation and biofilm formation	Chickpea	Qurashi and Sabri [61]
*Pseudomonas aeruginosa* PF07	Enhances root-adhering soil to root tissue ratio (RAS/RT)	Sunflower	Tewari and Arora [62]
*Rhodopseudomonas palustris* TN114 *Rhodopseudomonas palustris* PP803	Reduces Na^+^ in aqueous solution by EPS binding of Na^+^ (Na^+^ binding by galacturonic acid)		Nunkaew et al. [63]
*Bacillus amyloliquefaciens* SQR9	Improves Na^+^ homeostasis	Maize	Chen et al. [64]
*Bacillus amyloliquefaciens* HM6	Root protection by enhancing biofilm stability	Barley	Kasim et al. [65]
*Pseudomonas sp.* AK-1	Restricted Na^+^ influx due to free soil Na^+^ binding by EPS	Soybean	Kasotia et al. [54]
*Pseudomonas anguilliseptica* SAW24	Root protection by enhancing biofilm stability	Faba bean	Mohammed [66]
*Bacillus subtilis TP7* *Marinobacter lipolyticus SM19*	Restricted Na^+^ uptake	Wheat	Atouei et al. [67]

**Table 2:** PGPR’s mechanisms to alleviate drought stress in plants.

Bacteria	EPS Mechanism	Crop	Reference
*Bacillus polymyxa* CF43	Increases the mass of soil adhering to the roots	Wheat	Bezzate et al. [75]
*Pantoea agglomerans* NAS206	Augments the root-adhering soil (RAS) in both excessive or deficit water	Wheat	Amellal et al. [129]
*Rhizobium sp.* YAS34	Increases root-adhering soil (RAS) per root dry mass and soil macropore volume	Sunflower	Alami et al. [109]
*Rhizobium sp.* KYGT207	Enhances root-adhering soil (RAS) dry mass (dm) per root dm (RAS/RT) and RAS aggregate water stability	Wheat	Kaci et al. [136]
*Pseudomonas putida GAP-P45*	Improves soil aggregates stability	Sunflower	Sandhya et al. [71]
*Pseudomonas entomophila* BV-P13	Influence higher soil aggregates stability and mean weight diameter of root-adhering soil (RAS)	Maize	Sandhya et al. [120]
*Pseudomonas stutzei* GRFHAP-P14			
*Pseudomonas putida* GAP-P45			
*Pseudomonas syringae* GRFHYTP52			
*Pseudomonasmonteilli* WAPP53			
*Bacillus sp.* HYD-B17	Increase root-adhering soil (RAS) dry mass (dm) per root dm (RAS/RT) and weight diameter of soil aggregates	Maize	Sandhya et al. [14]
*Bacillus sp.* HYTAPB18			
*Bacillus sp.* HYDGRFB19			
*Bacillus sp.* BKB30			
*Bacillus sp.* RMPB44			
*Pseudomonas aeruginosa* B2	Augment soil aggregates stability	Maize	Putrie et al. [141]
*Brevibacillus brevis* B33			
*Proteus penneri* Pp1	Enhance moisture and water content of soil	Maize	Nassem and Bano [138]
*Pseudomonas aeruginosa* Pa2			
*Alcaligenes faecalis* AF3			
*Azotobacter sp.* AztRMD2	Augment soil aggregates stability	Rice	Sivapriya et al. [142]
*Bacillus amyloliquefaciens* FZB42	Plant protection by enhancing biofilm stability	*Arabidopsis thaliana*	Lu et al. [110]
*Pseudomonas aeruginosa* MCCB0035	Increase moisture and water content of soil	*Okra Plant*	Yadav et al. [143]
*Bacillus coagulans* MCCB0059			
*Planomicrobium chinese* P1	Maintain high moisture content in soil	Wheat	Khan and Bano [47]
*Bacillus cereus* P2			
